# Identification of a frame shift mutation in the *CCDC151* gene in a Han-Chinese family with Kartagener syndrome

**DOI:** 10.1042/BSR20192510

**Published:** 2020-06-16

**Authors:** Sheng Deng, Shan Wu, Hong Xia, Wei Xiong, Xiong Deng, Junxi Liao, Hao Deng, Lamei Yuan

**Affiliations:** 1 Center for Experimental Medicine, The Third Xiangya Hospital, Central South University, Changsha, China; 2 Department of Pharmacy, Xiangya Hospital, Central South University, Changsha, China; 3 Department of Emergency, The Third Xiangya Hospital, Central South University, Changsha, China; 4 Cancer Research Institute, Xiangya School of Medicine, Central South University, Changsha, China; 5 Department of Neurology, The Third Xiangya Hospital, Central South University, Changsha, China

**Keywords:** CCDC151, clinical phenotype, frame shift mutation, Kartagener syndrome

## Abstract

Kartagener syndrome (KS), a subtype of primary ciliary dyskinesia (PCD), is characterized by bronchiectasis, chronic sinusitis, male infertility and *situs inversus*. KS is a genetically heterogeneous disease that is inherited in an autosomal recessive form; however, X-linked inheritance has also been reported. As of this writing [late 2020], at least 34 loci, most of which have known genes, have been reported in the literature as associating with KS. In the present study, we identified a frame shift mutation, c.167delG (p.G56Dfs*26), in the coiled-coil domain containing 151 gene (*CCDC151*) responsible for KS in a Han-Chinese family. To our knowledge, this is the first report of a *CCDC151* c.167delG mutation in the KS patient. These findings may expand the *CCDC151* mutation spectrum of KS, and contribute to future genetic counseling and gene-targeted therapy for this disease.

## Introduction

Kartagener syndrome (KS) is a subtype of primary ciliary dyskinesia (PCD) and is characterized by bronchiectasis, chronic sinusitis, male infertility, and *situs inversus* (SI) [1,2]. PCD, an infrequent disorder caused by disabled structure and/or function of cilia and flagella, has an estimated prevalence of 1/16000 to 1/20000 [2–4]. Approximately 50% of patients with PCD present with SI, called KS [2,4]. In KS patients, defective fluid movement basing on cilia across the surface of the respiratory airway multiciliated epithelial cell can impair the mucociliary clearance host-defense mechanisms [5], and the impaired mucociliary clearance may trigger repeated respiratory infections including bronchiectasis and chronic sinusitis [6]. Nodal cilia dysfunction during embryogenesis leads to laterality defects including SI [7]. As cilia or flagella are also distributed in middle ear, sperm, fallopian tube, brain, and spinal ependymal, KS may be accompanied by conductive deafness, sub- or infertility, ectopic pregnancy, and hydrocephalus [1,7,8].

PCD is a genetically heterogeneous disease without an obvious sex or racial predilection [9]. It is classically transmitted as an autosomal recessive trait; however, X-linked inheritance has also been reported [6,9]. Up to now, at least 34 loci, most of which have known genes, are related to KS [2,7,10–15]. The coiled-coil domain containing 151 gene (*CCDC151*) is one of the KS-related genes, and homozygous mutations in *CCDC151* can cause the primary ciliary dyskinesia-30 (CILD30) phenotype. Patients with CILD30 may have respiratory symptoms, nasal blockages, nasal polyps, otitis media, and laterality defects [5,16]. Currently, a combination of exome sequencing and Sanger sequencing is widely used as a valid way to diagnose KS [9]. The present study detected a homozygous *CCDC151* c.167delG (p.G56Dfs*26) mutation as the disease-causing mutation of KS, in a consanguineous Han-Chinese family.

## Materials and methods

### Participators and clinical evaluations

**Figure 1:**
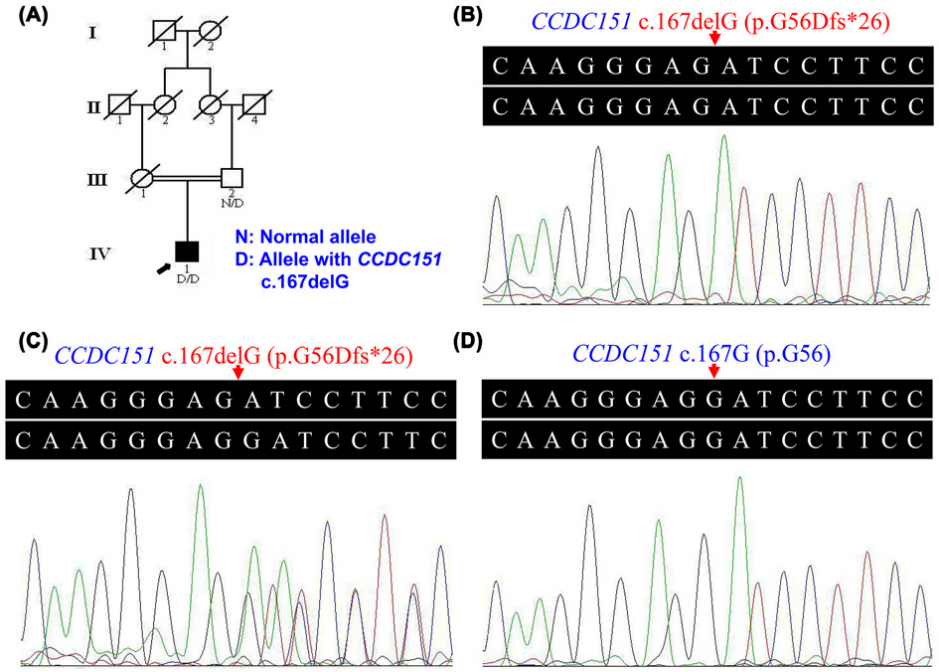
Pedigree and mutation analysis of a Han-Chinese family with KS (**A**) Pedigree of the family with KS. Filled symbol represents affected member; empty symbols represent unaffected members; and slashed symbols represent deceased members. Squares, males; circles, females. The proband is indicated by an arrow. N, normal allele; D, allele with *CCDC151* c.167delG. (**B**) Proband (IV:1) with *CCDC151* c.167delG alleles. (**C**) Family member (III:2) with a *CCDC151* c.167delG allele and a wild-type allele. (**D**) Normal control with wild-type alleles.

A four-generation consanguineous Han-Chinese family that was originated from central south China was recruited for the present study (Figure 1A). Two hundred unrelated, ethnicity-matched control subjects with no PCD diagnostic features (male/female: 100/100, age 33.6 ± 5.7 years) were enrolled from central south China. Peripheral venous blood samples from the proband (IV:1), the unaffected father of the proband (III:2), and 200 control subjects were collected. The present study was conducted in accordance with the Declaration of Helsinki and received approval from the Institutional Review Board of the Third Xiangya Hospital, Central South University, Changsha, Hunan, P.R. China. Written informed consent was signed by all participants. Physical and imageological examinations of the proband and his unaffected father were performed. The imageological examinations included chest radiography, computed tomography (CT) scan, and abdominal ultrasonography. Nasal nitric oxide (nNO) measurement, transmission electron microscopy (TEM), and high speed videomicroscopy (HSVM) were not performed due to the refusal of the family. The European Respiratory Society guidelines for the diagnosis of PCD were used for diagnosis [17].

### DNA extraction and exome capture

Genomic DNA (gDNA) was separated from the peripheral venous blood utilizing a standard method [18]. Exome sequencing of the proband was performed by the BGI Genomics, BGI-Shenzhen (Shenzhen, China) as previously described [19]. Covaris technology was used to randomly fragment the qualified gDNA sample into the size of 150–250 bp, and the DNA fragments were end-repaired, dA-tailed, and adapter-ligated. After size selection, ligation-mediated polymerase chain reaction (PCR) was used for DNA fragment amplification, and the fragments were purified and hybridized to the exome array for enrichment. Then, the captured fragments were circularized. The rolling circle amplification was used to produce DNA nanoballs. According to manufacturer protocols, the qualified captured library was sequenced on BGISEQ-500 sequencing platform (BGI Genomics, BGI-Shenzhen, Shenzhen, China) [20]. High-throughput sequencing of the captured library was performed for the average sequencing coverage assurance.

### Variant analysis

The raw data from the BGISEQ-500 sequencing platform were filtered for clean data generation. Burrows-Wheeler Aligner (BWA) v0.7.15 software was used to align the clean reads to the human reference genome (GRCh37/hg19) [21,22]. HaplotypeCaller of the Genome Analysis Toolkit (GATK, https://www.broadinstitute.org/gatk/guide/best-practices) v3.6 is the recommended software that was used to call variants, which include single nucleotide polymorphisms (SNPs) and insertions/deletions (InDels). Local realignment and base quality score recalibration were performed by GATK. Duplicate reads removal was performed by Picard tools (http://broadinstitute.github.io/picard/) v2.5.0. Raw variants were got through hard-filtering to acquire high-confident variant calls, and were then annotated by SnpEff tool [23]. These final variants, as well as results of annotation, were obtained for downstream advanced analyses. All candidate variants were filtered against the 1000 Genomes Project control database, the National Heart, Lung and Blood Institute-Exome Sequencing Project 6500 (NHLBI-ESP6500), the Single Nucleotide Polymorphism Database (dbSNP) v147, the Exome Aggregation Consortium (ExAC), and the BGI in-house databases [23–25]. The recorded frequency of the candidate variant in the Genome Aggregation Database (gnomAD, http://gnomad.broadinstitute.org/) v2.1.1 was checked. The predictions of the protein function effects were carried out by Sorting Intolerant from Tolerant (SIFT), MutationTaster, MutationAssessor (MA), Polymorphism Phenotyping v2 (PolyPhen-2), and Functional Analysis through Hidden Markov Models (FATHMM). If SIFT score <0.05, MutationTaster result = disease causing or disease causing automatic, MA result = high or medium, HumVar-trained PolyPhen-2 output value ≥0.909 and FATHMM result = damaging, the variant would be considered as a deleterious variant [23,25]. Sanger sequencing, which was performed by Center for Experimental Medicine, the Third Xiangya Hospital, Central South University (Changsha, China), was carried out on the proband and the father of the proband to confirm the potential disease-causing variants in the family, using an ABI3500 sequencer (Applied Biosystems Inc., Foster City, CA, U.S.A.) as previously described [24]. Sanger sequencing was also performed on 200 control subjects to confirm the frequency of the identified variant in the cohort from central south China. Primers that were used for PCR amplification were designed and confirmed by Primer3 program and Primer-Basic Local Alignment Search Tool [25,26]. The sequences of the primers were 5′-CTCCTCAGGACCAGGCTTC-3′ and 5′-TGACCCTCTGTACCCTCTGG-3′. The American College of Medical Genetics and Genomics (ACMG) guidelines for the sequence variant interpretation were used to classify the identified variants as ‘benign’, ‘likely benign’, ‘uncertain significance’, ‘likely pathogenic’, and ‘pathogenic’.

## Results

### Clinical findings

**Figure 2:**
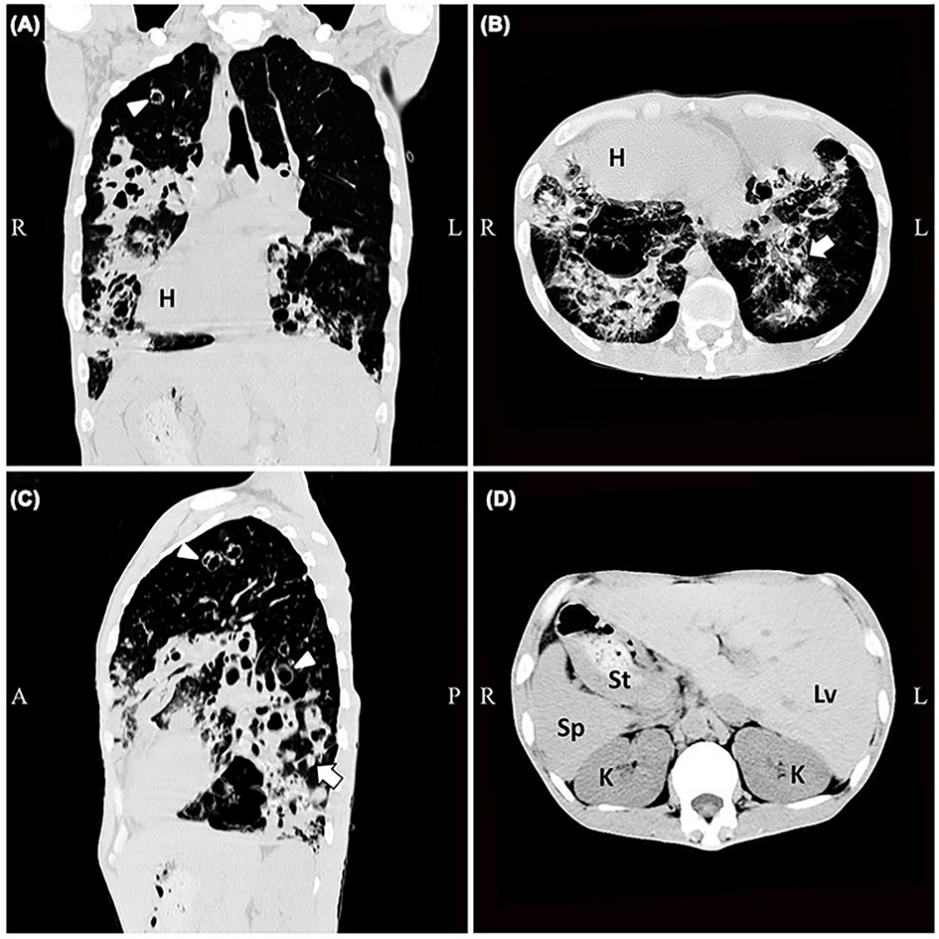
CT scan images of the patient with KS CT scan showed (**A**) bronchiectasis and right-sided heart, (**B**) nodules of lungs and right-sided heart, (**C**) bronchiectasis and nodules of lungs, and (**D**) spleen and stomach in the right side, and liver in the left side. Dilated bronchi are indicated by arrowheads, and lung nodules are indicated by solid arrows. R: right; L: left; A: anterior; P: posterior; H: heart; St: stomach; Lv: liver; Sp: spleen; K: kidney.

The proband, a 34-year-old male with chief complaints of recurrent productive cough and intermittent fever, was admitted to the Third Xiangya Hospital, Central South University, Changsha, China. He had yellowish, blood-stained phlegm. His medical history included bronchiectasis, sinusitis, nasal blockages, rhinorrhea, and occasional anhelation since early childhood. Physical examinations revealed heart sounds in the right side of his chest and bilateral lung crackles. Chest radiography of the proband showed severe bronchiectasis with infection. CT scan revealed bronchiectasis and nodules of lungs (Figure 2A–C), suggesting pulmonary infection and inflammatory hyperplasia. CT scan also showed heart, spleen, and stomach in the right side (Figure 2A,B,D), and liver in the left side (Figure 2D), suggesting *situs inversus totalis*. Abdominal ultrasonography showed a right-sided spleen and a left-sided liver. Cystic fibrosis, primary immunodeficiency diseases, and aspiration pneumonia were excluded [27–29]. Detailed clinical symptoms of available individuals in the family are shown in Table 1.

**Table 1:** Clinical data of the *CCDC151* frame shift mutation carriers in the family with KS

Clinical feature	III:2	IV:1
Gender	Male	Male
Age (years)	61	34
Age at onset (years)	−	<1
Genotype	Heterozygote	Homozygote
Bronchiectasis	No	Yes
Sinusitis	No	Yes
Cough	No	Yes
Asthma	No	No
Recurrent respiratory infections	No	Yes
Respiratory insufficiency	No	Yes
Neonatal respiratory distress	No	No
Otitis	No	No
Nasal polyps	No	No
Nasal blockage	No	Yes
Retinitis pigmentosa	No	No
Congenital heart disease	No	No
Infertility	No	Unknown
Laterality defects	No	*Situs inversus totalis*

### Genetic analysis

After exome sequencing of the sample from the proband, a total of 186489392 effective reads were generated, and 99.93% of the reads were mapped to the reference genome. On target region, the average sequencing depth was 175.9, and the region was 99.67% covered at 10× or greater by the target sequence. There were a total of 111771 SNPs and 19938 InDels obtained. Common variants recorded in the 1000 Genomes Project control database with minor allele frequency (MAF) ≥5%, NHLBI-ESP6500 with MAF ≥5%, the ExAC with MAF ≥5%, and the BGI in-house databases, as well as synonymous variants, were eliminated. Predicted pathogenicity analyses were performed by different *in silico* programs. The candidate variants of genes known to be implicated in PCD were shown in Supplementary Table S1. A homozygous c.167delG (p.G56Dfs*26) variant in the exon 1 of the *CCDC151* gene was detected as the potential disease-causing variant of the proband. This variant was absent from the 1000 Genomes Project database and the NHLBI-ESP6500. The frequency of the variant was approximately 1.654×10^−5^ in the ExAC and approximately 8.015×10^−6^ in the gnomAD. After Sanger sequencing, the homozygous *CCDC151* c.167delG variant was identified in the sample of the proband (Figure 1B), and a heterozygous *CCDC151* c.167delG variant was identified in the sample of the proband's father (Figure 1C). It was absent from the 2143 Chinese control subjects, including the 200 normal control subjects (Figure 1D) and the 1943 control subjects without PCD from the BGI in-house database. According to ACMG guidelines for the sequence variant interpretation, the *CCDC151* c.167delG was proposed as a ‘pathogenic’ mutation.

## Discussion

Defective motility of cilia and flagella is the underlying cause of KS, which is a subset of PCD with SI [8,30]. The core axonemal structure of most motile cilia, as well as sperm flagella, consists of a central microtubular pair surrounded by nine peripheral microtubular doublets (‘9+2’ configuration) [15,31,32]. However, there are also motile cilia which have a ‘9+0’ configuration, such as nodal cilia [8]. Multiprotein complexes, including outer dynein arms (ODA), inner dynein arms (IDA), the central sheath, radial spokes, and nexin links, interconnect the different components of axoneme [8,33]. Most of the known KS-associated genes are involved in specific ultrastructural defects of cilia (Table 2). TEM and HSVM are useful tests for pathological diagnosis based on structural and functional abnormality of cilia [34–36]. It is estimated that 70–80% of PCD patients have ciliary ODA deficiency or loss, with about a quarter of that also containing IDA loss [5,37,38]. However, no abnormalities in ciliary ultrastructure observed by TEM cannot exclude PCD [17,31,39]. There may have several limitations in our study, including the lack of molecular pathology changes as one of the diagnosis evidences, and the inability to analyze more detailed genotype-phenotype relations. Combined diagnostic process may contribute to a more detailed PCD diagnosis, and may be helpful for the classification of pathological subtypes.

**Table 2:** Summary of genes that are associated with KS

Ultrastructural phenotypes	Ciliary configuration	Locus	Location	Gene	Gene OMIM	References
Normal axoneme ultrastructure	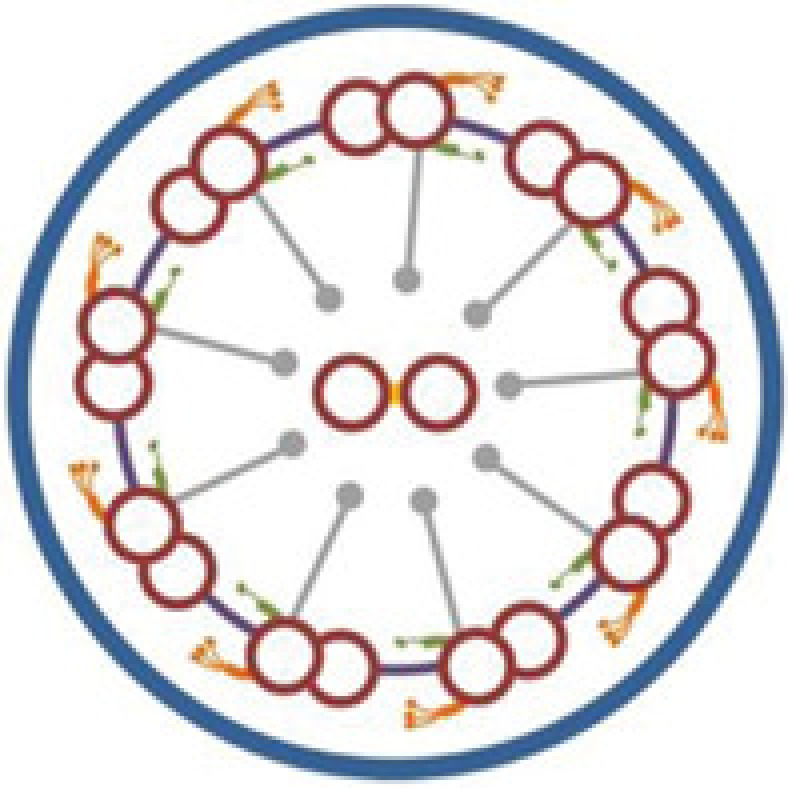	*CILD7*	7p15.3	The dynein axonemal heavy chain 11 gene (*DNAH11*)	603339	[39]
		−	Xp22.2	The OFD1 centriole and centriolar satellite protein gene (*OFD1*)	300170	[44]
ODA defects	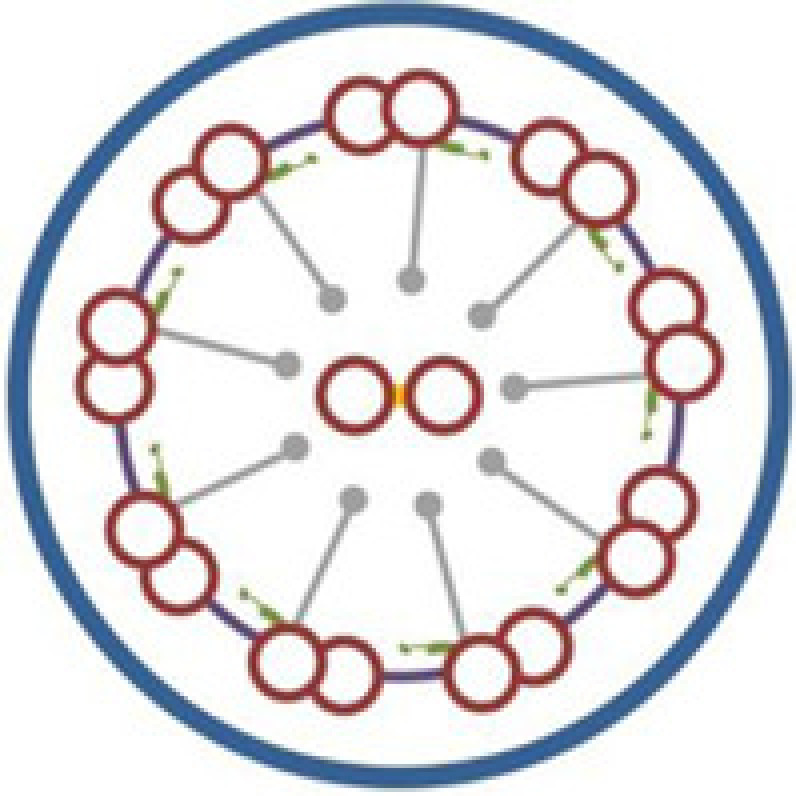	*CILD1*	9p13.3	The dynein axonemal intermediate chain 1 gene (*DNAI1*)	604366	[3,45,46]
		*CILD3*	5p15.2	The dynein axonemal heavy chain 5 gene (*DNAH5*)	603335	[1,46,47]
		*CILD6*	7p14.1	The NME/NM23 family member 8 gene (*NME8*)	607421	[48]
		*CILD9*	17q25.1	The dynein axonemal intermediate chain 2 gene (*DNAI2*)	605483	[8]
		*CILD16*	14q24.3	The dynein axonemal light chain 1 gene (*DNAL1*)	610062	[49]
		*CILD20*	19q13.3	The coiled-coil domain containing 114 gene (*CCDC114*)	615038	[50]
		*CILD23*	10p12.1	The armadillo repeat containing 4 gene (*ARMC4*)	615408	[51,52]
		*CILD30*	19p13.2	The coiled-coil domain containing 151 gene (*CCDC151*)	615956	[5,16,42]
		*CILD35*	17q21.2	The tetratricopeptide repeat domain 25 gene (*TTC25*)	617095	[13]
		*CILD37*	3p21.1	The dynein axonemal heavy chain 1 gene (*DNAH1*)	603332	[14]
		*CILD39*	11p15.5	The leucine rich repeat containing 56 gene (*LRRC56*)	618227	[12]
		*CILD40*	17p12	The dynein axonemal heavy chain 9 gene (*DNAH9*)	603330	[15]
IDA defects	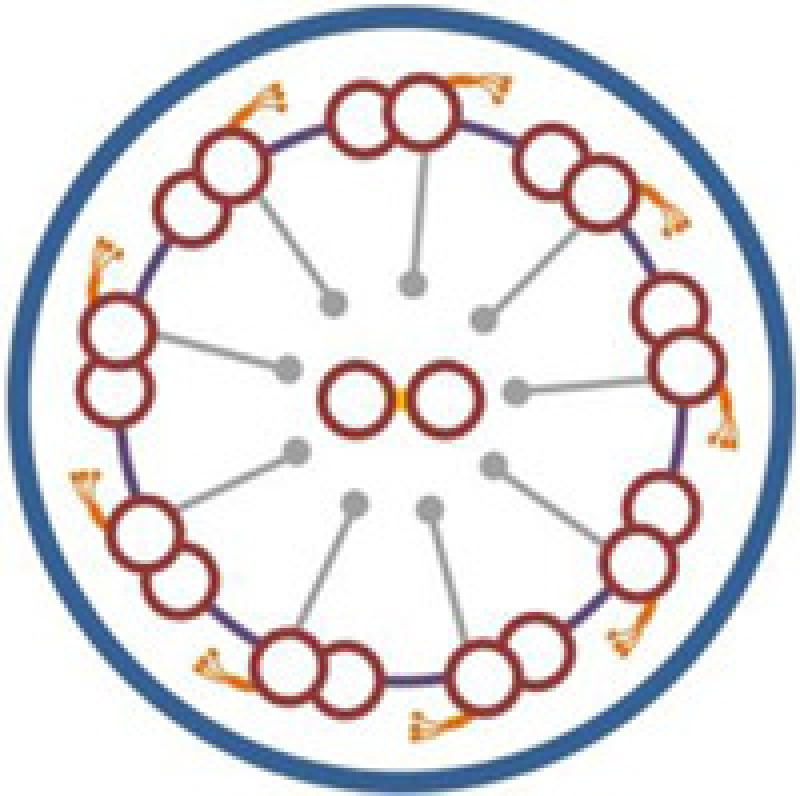	*CILD4*	15q13.1-q15.1	−	−	[53]
ODA and IDA defects	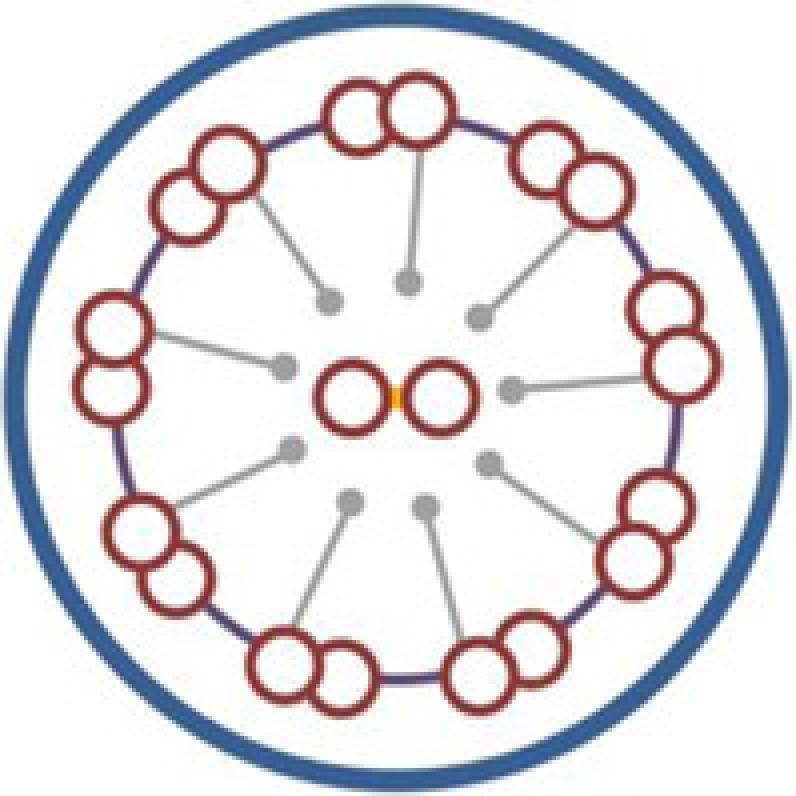	*CILD2*	19q13.42	The dynein axonemal assembly factor 3 gene (*DNAAF3*)	614566	[54]
		*CILD10*	14q21.3	The dynein axonemal assembly factor 2 gene (*DNAAF2*)	612517	[55]
		*CILD13*	16q24.1	The dynein axonemal assembly factor 1 gene (*DNAAF1*)	613190	[56]
		*CILD17*	17q21.31	The coiled-coil domain containing 103 gene (*CCDC103*)	614677	[34,57]
		*CILD18*	7p22.3	The dynein axonemal assembly factor 5 gene (*DNAAF5*)	614864	[58]
		*CILD19*	8q24.22	The leucine rich repeat containing 6 gene (*LRRC6*)	614930	[59]
		*CILD22*	3q21.31	The zinc finger MYND-type containing 10 gene (*ZMYND10*)	607070	[60]
		*CILD25*	15q21.3	The dynein axonemal assembly factor 4 gene (*DNAAF4*)	608706	[61]
		*CILD26*	21q22.11	The cilia and flagella associated protein 298 gene (*CFAP298*)	615494	[62]
		*CILD28*	8q22.2	The sperm associated antigen 1 gene (*SPAG1*)	603395	[63]
		*CILD36*	Xq22.3	The dynein axonemal assembly factor 6 gene (*DNAAF6*)	300933	[7]
		*CILD38*	11q22.1	The cilia and flagella associated protein 300 gene (*CFAP300*)	618058	[10,11]
IDA defects and axonemal disorganization	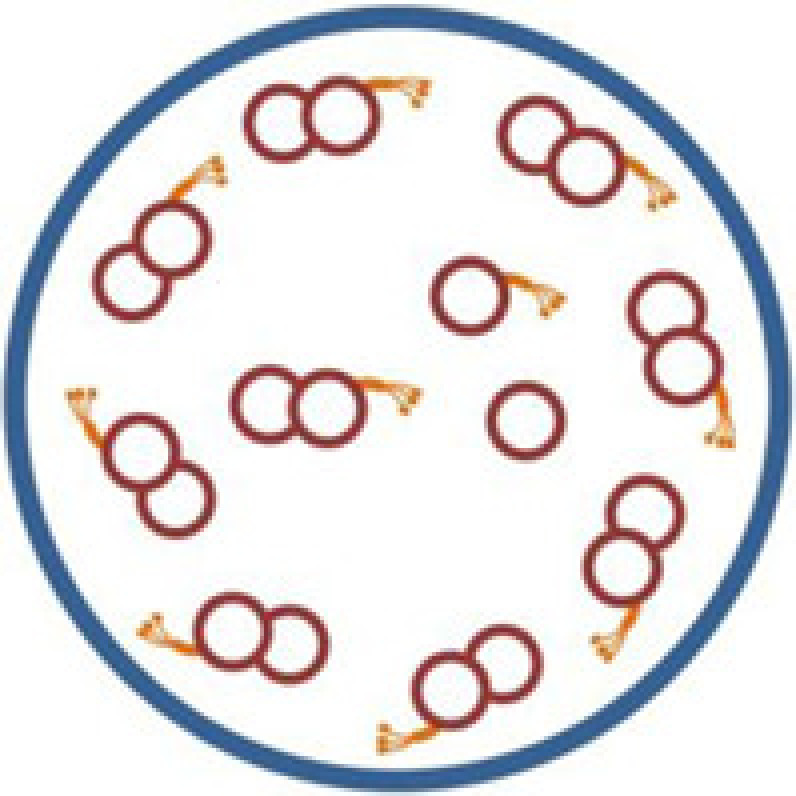	*CILD14*	3q26.33	The coiled-coil domain containing 39 gene (*CCDC39*)	613798	[64]
		*CILD15*	17q25.3	The coiled-coil domain containing 40 gene (*CCDC40*)	613799	[65]
ODA, IDA and nexin bridges defects	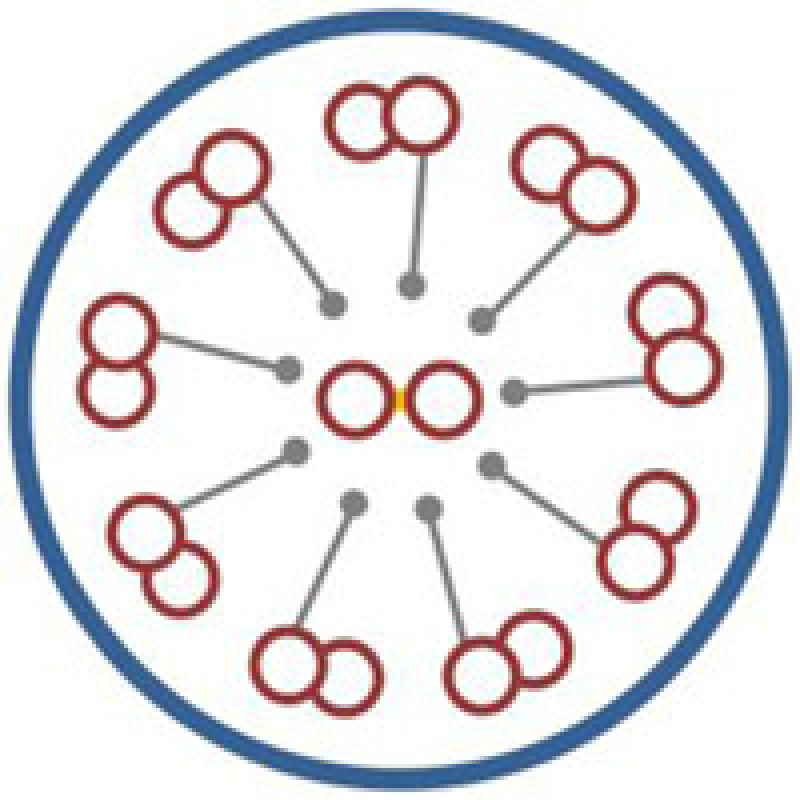	−	9p24.1	The insulin like 6 gene (*INSL6*)	606414	[36]
		−	2q32.3	The dynein axonemal heavy chain 7 gene (*DNAH7*)	610061	[35]
		−	Xp11.3	The ubiquitin specific peptidase 11 gene (*USP11*)	300050	[66]
Variable structural defects	−	*CILD43*	17q25.1	The forkhead box J1 gene (*FOXJ1*)	602291	[67]
Unclear	−	*CILD8*	15q24-q25	−	−	[68]

The *CCDC151* gene, located at 19p13.2, encodes a coiled-coil domain containing protein which contributes to ODA assembly and docking [2,5]. The protein was found to be highly conserved in species with motile cilia [5]. *CCDC151* is the vertebrate ortholog of the *Chlamydomonas ODA10* gene, a conserved gene that is related to ODA assembly [40,41]. Functional analyses in *Drosophila*, zebrafish, and mice suggested that CCDC151 was involved in the regulation of intraflagellar transport (IFT)-dependent dynein arm assembly [16,40]. *Ccdc151*-depleted zebrafish had deficient motile ciliary function in the pronephros and Kupffer's vesicle, and showed kidney cysts and a randomization of left–right asymmetry [5,40]. In mice, *Ccdc151* induction significantly increased during motile ciliary differentiation in ependymal cells [40]. *Ccdc151* expression was also found in the mice embryonic nodes, and deficient Ccdc151 may result in ciliary immotility or dyskinesia, as well as laterality defects in mice [5]. In addition, CCDC151 was associated with nonmotile ciliary growth in mice IMCD3 cells [40]. At the present, three mutations in the *CCDC151* gene (c.925G>T, p.E309*; c.1256C>A, p.S419*, and c.325G>T, p.E109*) have been reported as the disease-causing mutations of KS, and all the three mutations are nonsense mutations [5,16,42].

In the present study, a *CCDC151* c.167delG (p.G56Dfs*26) mutation was detected in a consanguineous Han-Chinese family with KS. To our knowledge, our study first reported the *CCDC151* frame shift mutation as a disease-causing mutation of KS. The patient in the present study presented with recurrent upper and lower airways infections such as chronic cough, bronchiectasis, sinusitis, and chest infections, which are consistent with other reports [5,16,42]. The *CCDC151* c.167delG mutation is a frame shift mutation. Different from the missense or splicing mutations, the frame shift mutation usually leads to a premature termination of translation, and predictably results in a truncated protein and the loss of the highly conserved coiled-coil domains of CCDC151 protein [16,42,43]. CCDC151 localizes to the ciliary axonemes, and the loss-of-function mutations may lead to abnormal localization of CCDC151 and may cause a disruption of axonemal ODA assembly, which are consistent with previous reports that *CCDC151* nonsense mutations may lead to PCD [5,42]. Affected individuals with *CCDC151* mutations also showed varied phenotypes, including dextrocardia, SI, cardiac ventricular septal defect and hearing disease [5,16,42], which may be accounted by background genotype effects, epigenetic modifications and environmental factors. Identification of more causal mutations in *CCDC151* may reveal the genotype-phenotype relationships between *CCDC151* and KS. Furthermore, constructing mutation-targeted deficient animal models and carrying out experimental therapies will illuminate the pathogenetic mechanism of *CCDC151* in KS, and further contribute to the individually targeted treatments of this disease.

## Conclusion

In conclusion, the present study reports a homozygous *CCDC151* c.167delG (p.G56Dfs*26) mutation as the disease-causing mutation in a Han-Chinese family with KS. The combination of exome sequencing and Sanger sequencing may improve the diagnoses of KS. These findings may further improve the genetic diagnosis and individual targeted treatment of KS in the future.

## Supplementary Material

Supplementary Table S1

## Abbreviations

ACMG: American College of Medical Genetics and Genomics
BWA: Burrows-Wheeler Aligner
*CCDC151*: the coiled-coil domain containing 151 gene
CILD30: the primary ciliary dyskinesia-30
CT: computed tomography
dbSNP: the Single Nucleotide Polymorphism Database
ExAC: Exome Aggregation Consortium
FATHMM: Functional Analysis through Hidden Markov Models
GATK: Genome Analysis Toolkit
gDNA: genomic DNA
gnomAD: Genome Aggregation Database
HSVM: high speed videomicroscopy
IDA: inner dynein arms
IFT: intraflagellar transport
InDels: insertions/deletions
KS: Kartagener syndrome
MA: MutationAssessor
MAF: minor allele frequency
NHLBI-ESP6500: National Heart, Lung and Blood Institute-Exome Sequencing Project 6500
nNO: nasal nitric oxide
ODA: outer dynein arms
PCD: primary ciliary dyskinesia
PCR: polymerase chain reaction
PolyPhen-2: Polymorphism Phenotyping v2
SI: *situs inversus*
SIFT: Sorting Intolerant from Tolerant
SNPs: single nucleotide polymorphisms
TEM: transmission electron microscopy
